# Metabolomics study of treatment response to conbercept of patients with neovascular age-related macular degeneration and polypoidal choroidal vasculopathy

**DOI:** 10.3389/fphar.2022.991879

**Published:** 2022-09-19

**Authors:** Yinchen Shen, Hanying Wang, Xiaoyin Xu, Chong Chen, Shaopin Zhu, Lu Cheng, Junwei Fang, Kun Liu, Xun Xu

**Affiliations:** ^1^ Department of Ophthalmology, Shanghai General Hospital, School of Medicine, Shanghai Jiao Tong University, Shanghai, China; ^2^ National Clinical Research Center for Eye Diseases, Shanghai, China; ^3^ Shanghai Key Laboratory of Ocular Fundus Diseases, Shanghai, China; ^4^ Shanghai Engineering Center for Visual Science and Photomedicine, Shanghai, China; ^5^ Shanghai Engineering Center for Precise Diagnosis and Treatment of Eye Diseases, Shanghai, China

**Keywords:** anti-vegf, neovascular age-related macular degeneration, polypoidal choroidal vasculopathy, metabolomics, treatment response

## Abstract

**Background:** Neovascular age-related macular degeneration (nAMD) and polypoidal choroidal vasculopathy (PCV) are major causes of blindness in aged people. 30% of the patients show unsatisfactory response to anti-vascular endothelial growth factor (anti-VEGF) drugs. This study aims to investigate the relationship between serum metabolome and treatment response to anti-VEGF therapy.

**Methods:** A prospective longitudinal study was conducted between March 2017 and April 2019 in 13 clinical sites in China. The discovery group were enrolled from Shanghai General Hospital. The validation group consisted of patients from the other 12 sites. Participants received at least one intravitreal injection of 0.5 mg anti-VEGF drug, conbercept, and were divided into two groups - responders and non-responders. Serum samples of both groups were processed for UHPLC-MS/MS analysis. We constructed principal component analysis (PCA) and partial least squares discriminant analysis (PLS-DA) models to investigate the metabolic differences between two groups using SIMCA-P. Area under curve (AUC) was calculated to screen the biomarkers to predict treatment response. Metabolites sub-classes and enriched pathways were obtained using MetaboAnalyst5.0.

**Results:** 219 eyes from 219 patients (nAMD = 126; PCV = 93) were enrolled. A total of 248 metabolites were detected. PCA and PLS-DA models of the discovery group demonstrated that the metabolic profiles of responders and non-responders clearly differed. Eighty-five differential metabolites were identified, including sub-classes of diacylglycerophosphocholines, lysophosphatidylcholine (LPC), fatty acids, phosphocholine, etc. Responders and non-responders differed most significantly in metabolism of LPC (*p* = 7.16 × 10^-19) and diacylglycerophosphocholine (*p* = 6.96 × 10^-17). LPC 18:0 exhibited the highest AUC, which is 0.896 with 95% confidence internal between 0.833 and 0.949, to discriminate responders. The predictive accuracy of LPC 18:0 was 72.4% in the validation group.

**Conclusions:** This study suggests that differential metabolites may be useful for guiding treatment options for nAMD and PCV. Metabolism of LPC and diacylglycerophosphocholine were found to affect response to conbercept treatment. LPC 18:0 was a potential biomarker to discriminate responders from non-responders.

## Graphical Abstract

AMD, age-related macular degeneration; PCV, polypoidal choroidal vasculopathy; PLS-DA, partial least squares discriminant analysis; LPC, lysophosphatidylcholine; PC, phosphocholine; ROC, operating characteristic curve; AUC, area under the receiver operating characteristic curve; vWF, von Willebrand Factor. Note: The protein structure of vWF is downloaded from PDB (https://www.rcsb.org/).
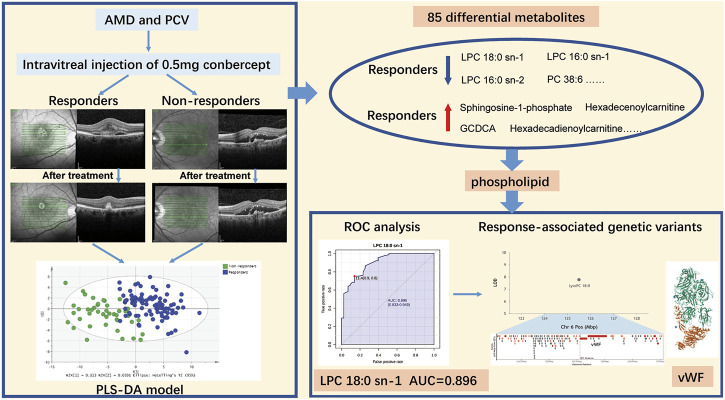

## Introduction

Neovascular age-related macular degeneration (nAMD) and polypoidal choroidal vasculopathy (PCV) are major causes of blindness in people worldwide over 50 years old. More than 20% of the ageing population may suffer from the disorders (Lim et al., 2012). The Global Burden of Disease Study 2010 reported an increase of 160% in years lived with disability related to AMD (Vos et al., 2012). nAMD, characterized by the subfoveal choroidal neovascularization (CNV), have been found to cause structural damage to the macular and lead to irreversible central vision impairment or even blindness within a few years (Rastoin et al., 2020). PCV, a subtype of nAMD, however, is thought to potentially have different pathogenic mechanisms. It is mostly found in Asian population, manifested as orange-red nodular lesions (Ho et al., 2018).

The common etiology of nAMD and PCV is due to the pathological angiogenesis mediated by the vascular endothelial growth factor (VEGF) (Rastoin et al., 2020). Therefore, intravitreal anti-VEGF therapy is recommended as the first-line treatment option for this kind of disease (Campa et al., 2011; Cheung et al., 2018; Toto et al., 2021). Despite the efficacy of anti-VEGF drugs, real-world outcomes were found to be less favorable than the results of randomized-controlled clinical trials (Schmidt-Erfurth et al., 2014). Anti-VEGF drugs only delayed the progression to blindness, and about 30% of the patients show unsatisfactory response to treatment (Rastoin et al., 2020; Mettu et al., 2021). Recent studies demonstrated that the inter-individual differences in treatment response depended on a variety of factors, such as age, lifestyles, anatomical structure of the lesions, genetic polymorphism, etc (Amoaku et al., 2015). Although intravitreal injection has been proved to be safe, intraocular infection remains a severe and destructive complication (Storey et al., 2020). In the developing countries, the economic burden of multiple anti-VEGF treatments is also an unavoidable social problem.

For the reasons above, it is urgent to develop effective methods to predict response to anti-VEGF therapy and to personalize treatment options. Metabolomics studies are developed to uncover metabolic alteration in response to external or internal subtle perturbation. As metabolism is located at the end of life activities, metabolomics can reflect the changes that have occurred in the organism. Therefore, metabolomics data can be more easily correlated with clinical phenotypes. Dysregulation of lipid and metabolic pathways were found to play important roles in the development of AMD (Hou et al., 2020) and PCV (Li et al., 2016). Nevertheless, the serum lipid and metabolic differences between responders and non-responders remained unclear. We believe that there is a great need to better understand response to anti-VEGF therapy and that metabolomics is a potential area for addressing it. Therefore, the aim of this study is to conduct metabolic profiling to identify reliable serum biomarkers to discriminate responders to anti-VEGF treatment.

## Materials and methods

### Study design

**FIGURE 1:**
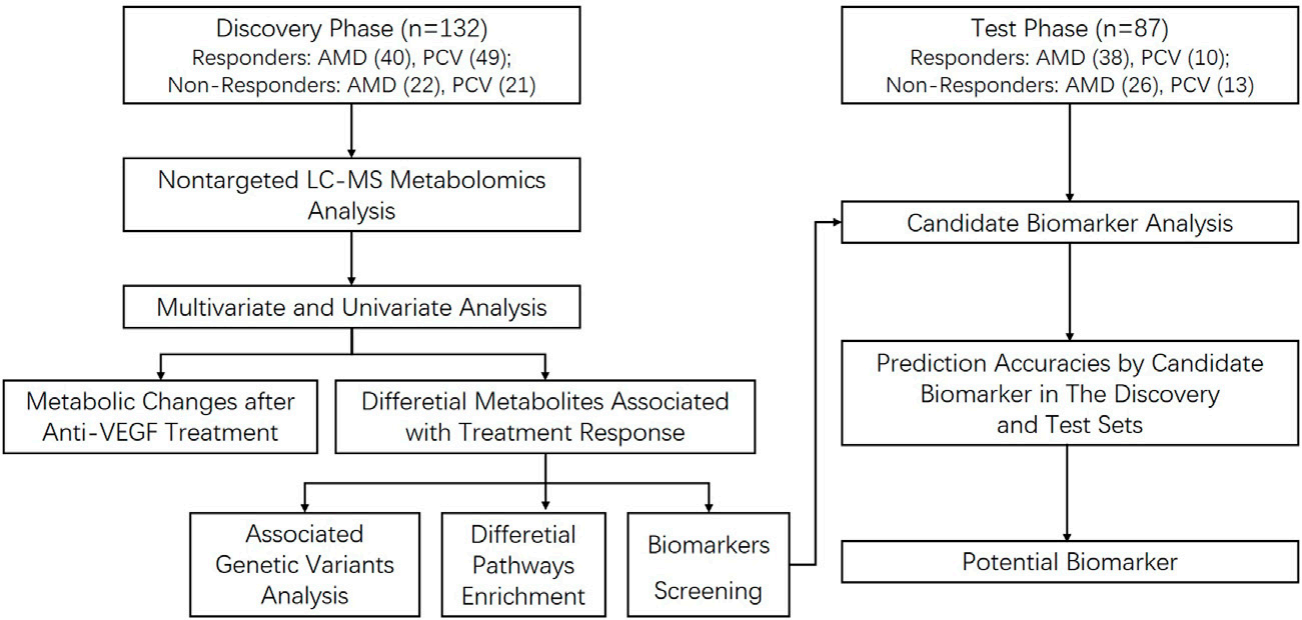
The procedure of the study.

A prospective longitudinal study was conducted between March 2017 and April 2019 in 13 clinical sites in China. The discovery group were enrolled from Department of Ophthalmology, Shanghai General Hospital, School of medicine, Shanghai Jiao Tong University, Shanghai, China, from 13 March 2017 to 1 March 2019. The last patient last visit (LPLV) of the discovery group was on 1 April 2019. The validation group consisted of patients from another 12 clinical sites in mainland China, from 28 April 2017 to 20 December 2018. The LPLV of the validation group was on 21 January 2019. Figure 1 shows the procedure of the study. This study was approved by the ethics committee of Shanghai General Hospital (permit No. 2016KY115-2), in accordance with the Declaration of Helsinki, and the other clinical sites also obtained the ethical approval. This study was registered on www.ClinicalTrial.gov (NCT03128463). All subjects provided written consent forms. The study protocol has been published (Jin et al., 2018), and here we report the metabolic analysis results.

### Study subjects

The inclusion criteria were: (i) Age ≥50 years; (ii) Diagnosed as nAMD or PCV, and the diagnosis criteria was according to the guideline of Chinese Ocular Fundus Disease Society (The Clinical Guideline and Clinical Pathway Development Committee of Age-Related Macular Degeneration, Ocular Fundus Diseases Society, Chinese Ophthalmological Society, Chinese Medical Association, 2013); (iii) Received at least one intravitreal injection of conbercept (Lumitin; Chengdu Kanghong Biotech Co., Ltd., Chengdu, China). The exclusion criteria included: (i) Intravitreal or systemic administration of anti-VEGF drugs within 3 months; (ii) Other interventional therapies within 3 months (e.g. photodynamic therapy, retinal photocoagulation, pars plana vitrectomy, etc); (iii) Other reasons that caused subretinal of intraretinal fluid/hemorrhage, such as diabetic retinopathy, retinal venous occlusion, etc.; (iv) Serious systemic diseases, such as renal failure.

At Visit 1 (V1), all participants underwent comprehensive ophthalmologic examinations, including slit-lamp biomicroscopy, best-corrected visual acuity (BCVA) using standard Early Treatment Diabetic Retinopathy Study (ETDRS) letters, intraocular pressure (IOP) measurement, color fundus photography, and spectral-domain optical coherence tomography (SD-OCT) (Spectralis; Heidelberg Engineering, Heidelberg, Germany). All OCT scans were centered on the fovea, using a centrally oriented internal fixation mark. Central retinal thickness (CRT) within 1 mm of the central fovea were calculated automatically by the instrument. Fundus fluorescein angiography and indocyanine green angiography were also performed for the differential diagnosis of nAMD and PCV, except for the participants who had a history of allergy. The retinal specialists confirmed the diagnosis based on the fundus and morphology examinations, and then they administered intravitreal injections of 0.5 mg conbercept in the operation room under topical anesthesia. One month after treatment, a routine follow-up Visit 2 (V2) was performed. The results of BCVA, IOP, and SD-OCT were collected in V2.

### Grading of the response to Anti-VEGF therapy

In the discovery group, grading of response was based on the morphology changes on SD-OCT scans from V1 to V2, such as reduction in intraretinal fluid (IRF), subretinal fluid (SRF), and retinal thickening, independently judged by two experienced graders (Yinchen Shen, Hanying Wang). The grading was confirmed by a senior retinal specialist (Xun Xu). In case of discrepancy, a final agreement was reached by all the graders. Responders were defined as patients with a significant reduction in IRF or SRF and retinal thickening. Non-responders were defined as patients with an increase or no change in IRF, SRF, and retinal thickening, from V1 to V2 (Amoaku et al., 2015). In the validation group, responders were defined as patients with a reduction of CRT ≥10% of the baseline values, while non-responders were those with a reduction of CRT <10% of the baseline values or an increase of CRT 1 month after one injection of conbercept.

### Collection and pretreatment of serum samples

10 ml patients’ peripheral blood was obtained fasting in the morning before anti-VEGF injection at V1 and at V2, respectively, then was centrifuged within 30 min (1,500 rpm, 10 min at 20°C). The serum aliquots were collected and stored at −80°C immediately for further analysis. The procedures to extract metabolites were as follows: 100 μL sample was extracted by 4-fold volume methanol extraction agent. The mixture was then vortexed and centrifuged. 180 μL lyophilized samples were used for positive and negative ion analysis, respectively. Before analysis, the lyophilized samples were re-dissolved with 50 μl 25% acetonitrile, and the system was balanced with blank samples. We carried quality control (QC) to monitor the stability and repeatability of the analysis. The QC samples were prepared the same way by pooling equal volume of all serum samples, injected after every ten samples.

### Metabolomics profiling

The samples were processed in the same batch for UHPLC-MS/MS analysis on a hybrid quadrupole-Orbitrap mass spectrometer (Vanquish UPLC-Q Exactive, Thermo Fisher Scientific, Rockford, IL, USA). For positive ion mode, we used Waters BEH C8 column (50 mm × 2.1 mm, 1.7 μm) (Waters, Milford, MA) to separate mixture at 60°C. Phase A: water with 0.1% formic acid; phase B: acetonitrile with 0.1% formic acid. Flow rate was set at 0.4 ml/min with the following gradient: 0–0.5 min, 5%B; 0.5–2 min, linearly increased to 40%B; 2–8 min, linearly increased to 100% B; maintained for 2 min; 10.1 min, decreased back to 5% B; balanced for 2 min. For negative ion mode, we used ACQUITY UPLC HSS T3 (50 mm × 2.1 mm, 1.8 μm) (Waters, Milford, MA) to separate mixture at 60°C. Phase A: 6.5 mm NH4HCO3 added to water; phase B: 95% methanol and 6.5 mM NH4HCO3 aqueous solution. Flow rate was set at 0.4 ml/min with the following gradient: 0–0.5 min, 2%B; 0.5–2 min, linearly increased to 40%B; 2–8 min, linearly increased to 100% B; maintained for 2 min; 10.1 min, decreased back to 2% B; balanced for 1.9 min. The acquisition setting for metabolomics data: full scan mode; capillary temperature, 300°C; sheath gas, 45; auxiliary gas, 10; mass resolution, 7e4. For positive ions: spray voltage, 3.50 kV; m/z range, 80–1,200. For negative ions: spray voltage, 3.00 kV; m/z range, 80–1,200.

### Statistical analysis

The descriptive statistics were performed with SPSS software version 22.0 (SPSS Inc. Chicago, IL, United States). Continuous variables were expressed as mean (95% confidence internal). Comparisons of patients’ age, BCVA, CRT, IOP were performed using independent-samples t-tests or Mann-Whitney U. Categorical variables were expressed as number (percentage). Comparisons of patients’ gender, ethnicity, study eye, lesion type, systemic diseases, and medicine treatments were analyzed by Chi-square tests. Meanwhile, the weighted kappa for inter-observer imaging grading was calculated to verify the reliability and accuracy of the judgement. A *p* value <0.05 was considered statistically significant.

For metabolomics analysis, we constructed principal component analysis (PCA) and partial least squares discriminant analysis (PLS-DA) models to investigate the differences of metabolic profiles between responders and non-responders using SIMCA-P version 14.0 (Umetrics AB, Umea, Sweden). Fold change (FC) and false discovery rate (FDR) were calculated to reveal the metabolites with significant differences. The metabolites with FC > 1.2 and FDR <0.01 were defined as differential metabolites. Area under the receiver operating characteristic curve (AUC) was calculated to screen the biomarkers to discriminate responders. In order to verify the potential biomarker, its optimal cut-off value of the discovery group was employed with the validation group to test the prediction accuracy. Volcano plot, venn plot, interactive pie chart of metabolites sub-classes, and bar plot of enriched pathways were drawn using MetaboAnalyst5.0 (http://www.metaboanalyst.ca/). We created a Github page and upload all related scripts and supported data. The link of the Github page is https://github.com/epang2022/Metabolomics-Study-of-Treatment-Response-to-Conbercept-of-Patients-with-Neovascular-Age-related-Macu .

### Association analysis of Anti-VEGF response - Associated genetic variants with metabolites

In order to reveal possible genetic variants related to identified metabolites in this study, a public platform Lipid Genie’s Lipid Viewer (http://www.lipidgenie.com/qtlViewer.html) was used to explore genome-metabolite connections. After selecting a specific lipid species, the Logarithm-of-Odds (LOD) and the allele effect plots for mapped Quantitative Trait Loci (QTLs) were visualized in parallel to genes located within a 3 megabase pair window of QTL’s apex LOD score.

## Result

In total, 219 eyes from 219 patients (nAMD, n = 126; PCV, n = 93) were enrolled. The discovery group included 132 eyes from 132 patients (nAMD, n = 62; PCV, n = 70). Another 87 independent subjects (nAMD, n = 64; PCV, n = 23) were recruited as the validation group. A total of 248 metabolites were detected by untargeted LC-MS metabolomics analysis, and the reproducibility of analysis was confirmed by QC samples. The relative standard deviations of 226 (91.1%) metabolites were less than 30%.

### Study populations

The clinical characteristics are in Table 1. There were no statistical differences in age, gender, ethnicity, study eye, baseline BCVA, and baseline CRT between responders and non-responders for both populations. The weighted kappa for inter-observer imaging grading of the discovery set was 0.862 (*p* < 0.001). In the discovery group, the mean CRT of responders reduced significantly from 502.8 (458.8–546.8) μm to 342.9 (315.0–370.8) μm, *p* < 0.001. However, CRT slightly increased from 524.5 (451.2–597.8) μm to 530.3 (455.0–605.7) μm for non-responders, *p* = 0.348. There were also more gains in BCVA for responders. For the validation group, CRT of responders decreased from 397.5 (351.8–443.3) μm to 260.7 (233.3–288.2) μm, *p* < 0.001. Nevertheless, CRT increased slightly for non-responders, from 348.9 (304.0–393.7) μm to 364.7 (319.1–410.4) μm, *p* = 0.076. BCVA gains for responders did not reach statistical significance.

**TABLE 1:** Clinical characteristics of study populations.

	Discovery group			Validation group		
	Responders (n = 89)	Non-responders (n = 43)	*p*-value	Responders (n = 48)	Non-responders (n = 39)	*p*-value
Age, years, mean (CI) ^a^	69.6 (67.8–71.4)	68.5 (66.6–70.4)	0.408	69.0 (66.6–71.4)	67.3 (65.0–69.5)	0.306
Gender, male, n (%)^b^	60 (67.4)	32 (74.4)	0.412	33 (68.8)	26 (66.7)	0.836
Ethnicity, Han, n (%)^b^	89 (100.0)	41 (95.3)	0.103	48 (100.0)	39 (100.0)	1.000
Study eye, right, n (%)^b^	53 (59.6)	24 (55.8)	0.683	22 (45.8)	20 (51.3)	0.613
Lesion type, AMD, n (%)^b^	40 (44.9)	22 (51.2)	0.502	38 (79.2)	26 (66.7)	0.189
**Clinical characteristics**						
BCVA before treatment, ETDRS letters, (CI) ^a^	39.0 (34.1–43.9)	42.2 (35.4–49.0)	0.462	41.9 (35.6–48.3)	43.8 (35.7–51.9)	0.710
BCVA after treatment, ETDRS letters, (CI) ^c^	46.3 (41.5–51.2)	44.4 (37.0–51.7)	0.638	48.7 (42.6–54.9)	49.0 (41.7–56.3)	0.980
Change of BCVA, ETDRS letters, (CI) ^c^	7.3 (4.7–10.0)	2.2 (-0.5–4.9)	0.008	6.8 (2.8–10.9)	5.2 (0.84–9.6)	0.484
IOP before treatment, mmHg, (CI)	14.64 (13.95–15.34)	15.21 (14.31–16.11)	0.236^c^	13.88 (12.90–14.86)	14.62 (13.52–15.72)	0.312^a^
IOP after treatment, mmHg, (CI) ^c^	13.93 (13.35–14.51)	13.95 (13.18–14.73)	0.965	12.95 (11.98–13.91)	14.32 (13.28–15.36)	0.047
Change of IOP, mmHg, (CI)	-0.71 (-1.34∼-0.09)	-1.26 (-1.86∼-0.65)	0.082^c^	-0.93 (-1.66∼-0.20)	-0.30 (-1.11–0.51)	0.245^a^
CRT before treatment, μm, (CI) ^c^	502.8 (458.8–546.8)	524.5 (451.2–597.8)	0.928	397.5 (351.8–443.3)	348.9 (304.0–393.7)	0.069
CRT after treatment, μm, (CI) ^c^	342.9 (315.0–370.8)	530.3 (455.0–605.7)	<0.001	260.7 (233.3–288.2)	364.7 (319.1–410.4)	<0.001
Change of CRT, μm, (CI) ^c^	-159.9 (-187.1∼-132.7)	5.9 (-5.8–17.5)	<0.001	-136.8 (-167.0∼-106.7)	15.9 (-1.7–33.5)	<0.001
**Systemic diseases at baseline, n (%)** ^b^						
Hypertension	29 (32.6)	18 (41.9)	0.297	13 (27.1)	13 (33.3)	0.527
Diabetes	14 (15.7)	3 (7.0)	0.259	8 (16.7)	3 (7.7)	0.353
Dyslipidemia	5 (5.6)	3 (7.0)	1.000	0 (0.0)	2 (5.1)	0.198
Cardiac/cerebrovascular diseases	11 (12.4)	3 (7.0)	0.522	0 (0.0)	2 (5.1)	0.198
**Treatments, n (%)** ^b^						
Antihypertensives	24 (27.0)	15 (34.9)	0.350	8 (16.7)	14 (35.9)	0.040
Antihyperglycemics	12 (13.5)	2 (4.7)	0.214	6 (12.5)	1 (2.6)	0.194
Antihyperlipidemics	3 (3.4)	4 (9.3)	0.312	1 (2.1)	0 (0.0)	1.000

BCVA, best corrected visual acuity; ETDRS, early treatment diabetic retinopathy study; IOP, intraocular pressure; CRT, central retinal thickness.

a : Independent-samples *t* test.

b : Chi-square test.

c : Mann-Whitney *U* test.

### Comparison of metabolic profiles before and after Anti-VEGF treatment

**FIGURE 2:**
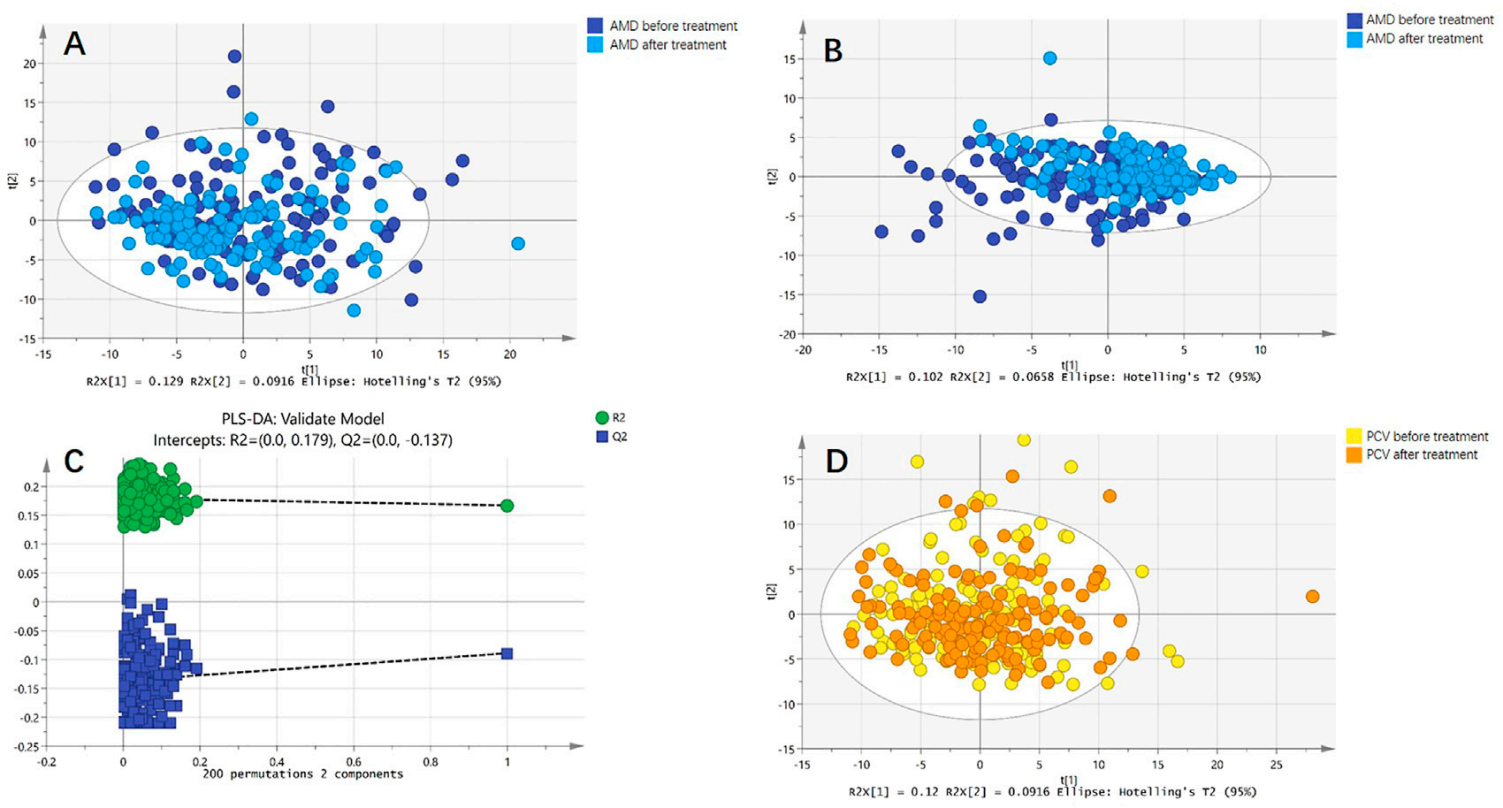
Comparison of metabolic profiles before and after anti-VEGF treatment. **(A)** Principal component analysis (PCA) model of patients with nAMD before and after anti-VEGF treatment. The PCA model showed no difference. **(B)** Partial least squares discriminant analysis (PLS-DA) model of patients with nAMD before and after anti-VEGF treatment. The PLS-DA model also showed no difference. **(C)** The permutation test indicated that PLS-DA model of nAMD overfitted. **(D)** PCA model of patients with PCV before and after anti-VEGF treatment. The PCA model showed no difference.

Both PCA and PLS-DA models were constructed for serum metabolic profiles of patients with nAMD before and after treatment. Neither model showed significant differences. The permutation test indicated that PLS-DA model of nAMD overfitted. Meanwhile, PCA model was constructed for patients with PCV before and after treatment, indicating no difference. PLS-DA model of PCV could not be established because of insignificant difference (Figure 2).

### Metabolic differences between responders and non-responders to Anti-VEGF treatment

**FIGURE 3:**
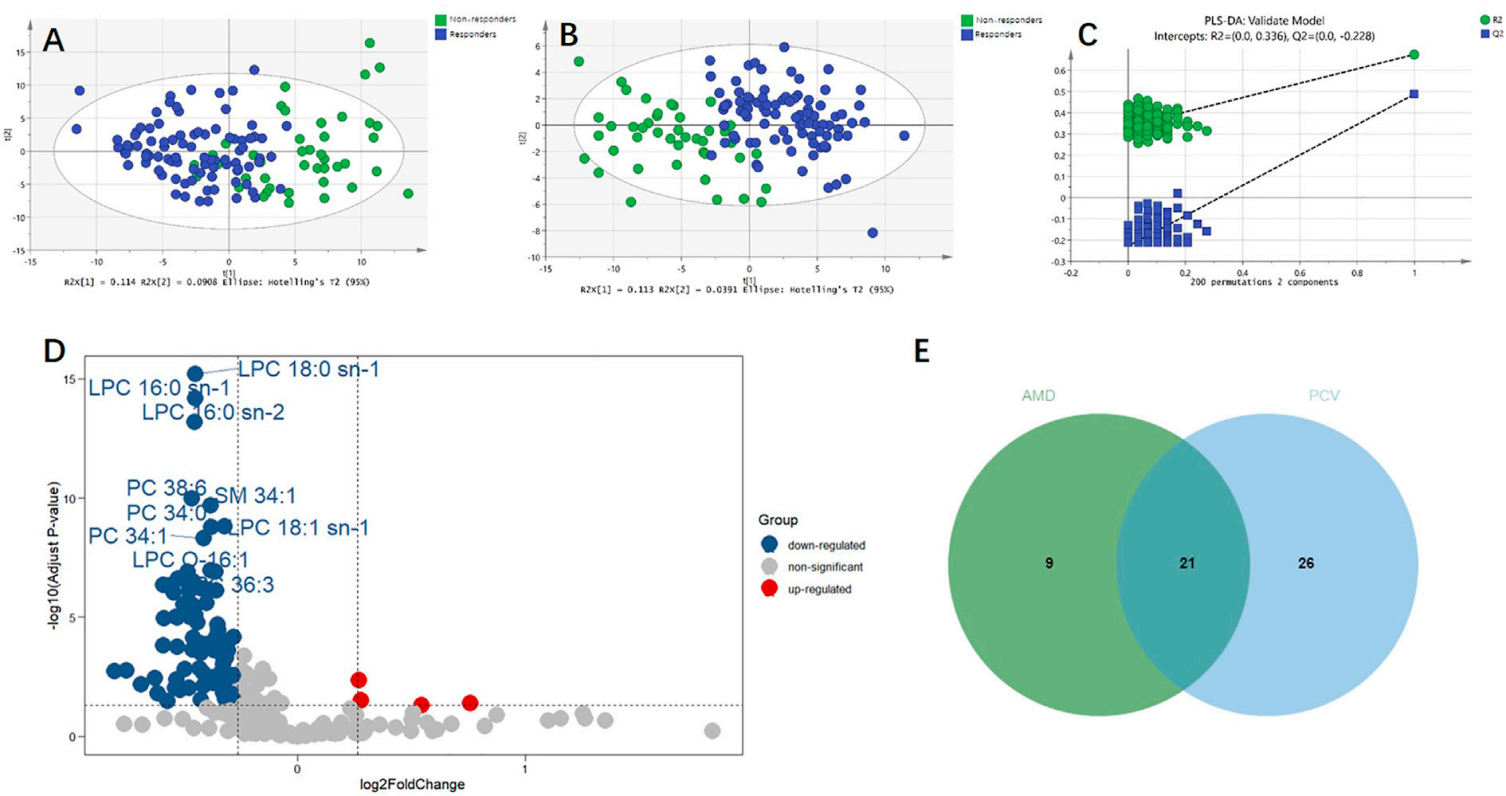
Metabolic differences between responders and non-responders to anti-VEGF treatment combining nAMD and PCV. **(A)** Principal component analysis (PCA) model showed significant difference. **(B)** Partial least squares discriminant analysis (PLS-DA) model showed significant difference. **(C)** The permutation test demonstrated that PLS-DA model did not overfit. **(D)** Volcano plot of the differential metabolites between responders and non-responders. Blue and red dots indicated down-regulated and up-regulated metabolites, respectively. **(E)** Venn plot of the differential metabolites between responders and non-responders in AMD (30 metabolites) and the differential metabolites between responders and non-responders in PCV (47 metabolites).

**FIGURE 4:**
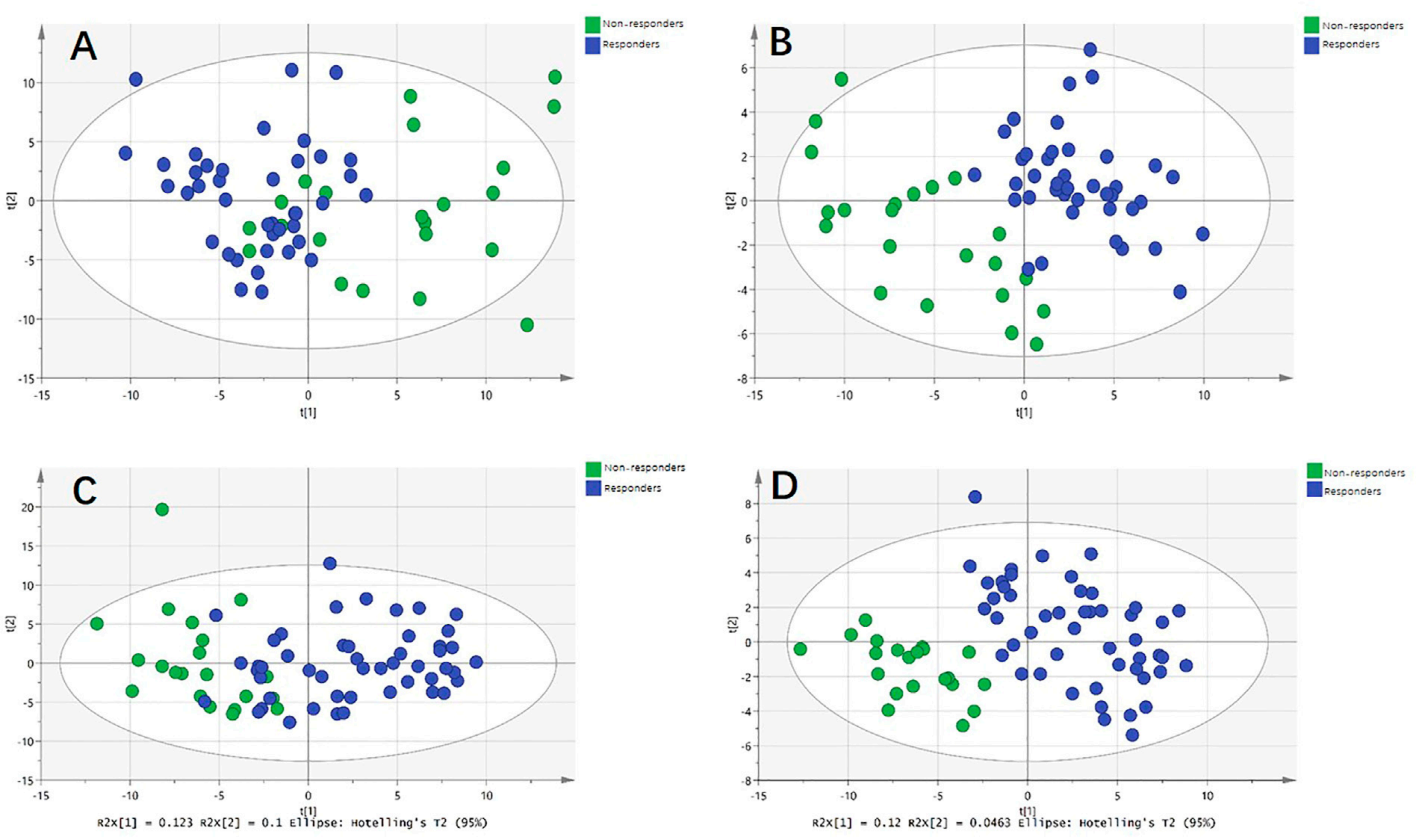
Metabolic differences between responders and non-responders to anti-VEGF treatment in nAMD and PCV, respectively. **(A)** Principal component analysis (PCA) model showed significant difference in nAMD. **(B)** Partial least squares discriminant analysis (PLS-DA) model showed significant difference in nAMD. **(C)** PCA model showed significant difference in PCV. **(D)** PLS-DA model showed significant difference in PCV.

For the discovery group, PCA and PLS-DA models both demonstrated that the baseline metabolic profile of responders and that of non-responders showed significant differences combining nAMD and PCV (Figure 3). In all, eighty-five differential metabolites were identified (see Supplementary Table S1). Table 2 lists the top 20 metabolites ranked by AUC. In addition, PCA and PLS-DA models were established for patients with nAMD and PCV, respectively. We also identified a clear separation between responders and non-responders in these models (Figure 4).

**TABLE 2:** Top 20 differential metabolites between responders and non-responders ranked by the area under the receiver operating characteristic curve (AUC).

No.	Differential metabolites	AUC	FDR	Log2 FC
1	LPC 18:0 sn-1	0.896	2.650 × 10^-18	0.451
2	LPC 16:0 sn-1	0.892	5.321 × 10^-17	0.449
3	LPC 16:0 sn-2	0.876	8.154 × 10^-16	0.454
4	PC 38:6	0.832	1.706 × 10^-12	0.465
5	SM 34:1	0.829	4.252 × 10^-12	0.383
6	LPC 18:1 sn-1	0.827	3.679 × 10^-11	0.323
7	PC 34:0	0.819	4.700 × 10^-11	0.380
8	PC 34:1	0.813	1.561 × 10^-10	0.415
9	PC 38:5	0.798	3.192 × 10^-8	0.477
10	PC 36:3	0.795	4.843 × 10^-9	0.363
11	PC O-38:6	0.785	4.852 × 10^-8	0.414
12	PC 36:2	0.784	5.403 × 10^-9	0.482
13	LPE 22:6 sn-1	0.784	1.147 × 10^-8	0.525
14	PC O-38:5	0.780	6.600 × 10^-8	0.357
15	LPC 18:2 sn-1	0.779	5.813 × 10^-8	0.390
16	PE O-38:7	0.778	2.551 × 10^-8	0.589
17	PC O-36:5	0.777	4.371 × 10^-8	0.426
18	LPC O-16:1	0.772	3.866 × 10^-9	0.382
19	PC 38:4	0.770	3.016 × 10^-7	0.466
20	LPC 16:1 sn-1	0.767	1.701 × 10^-8	0.459

AUC, area under the receiver operating characteristic curve; FDR, false discovery rate; FC, fold change.

### Enriched metabolite pathways and potential biomarker associated with different response to Anti-VEGF treatment

**FIGURE 5:**
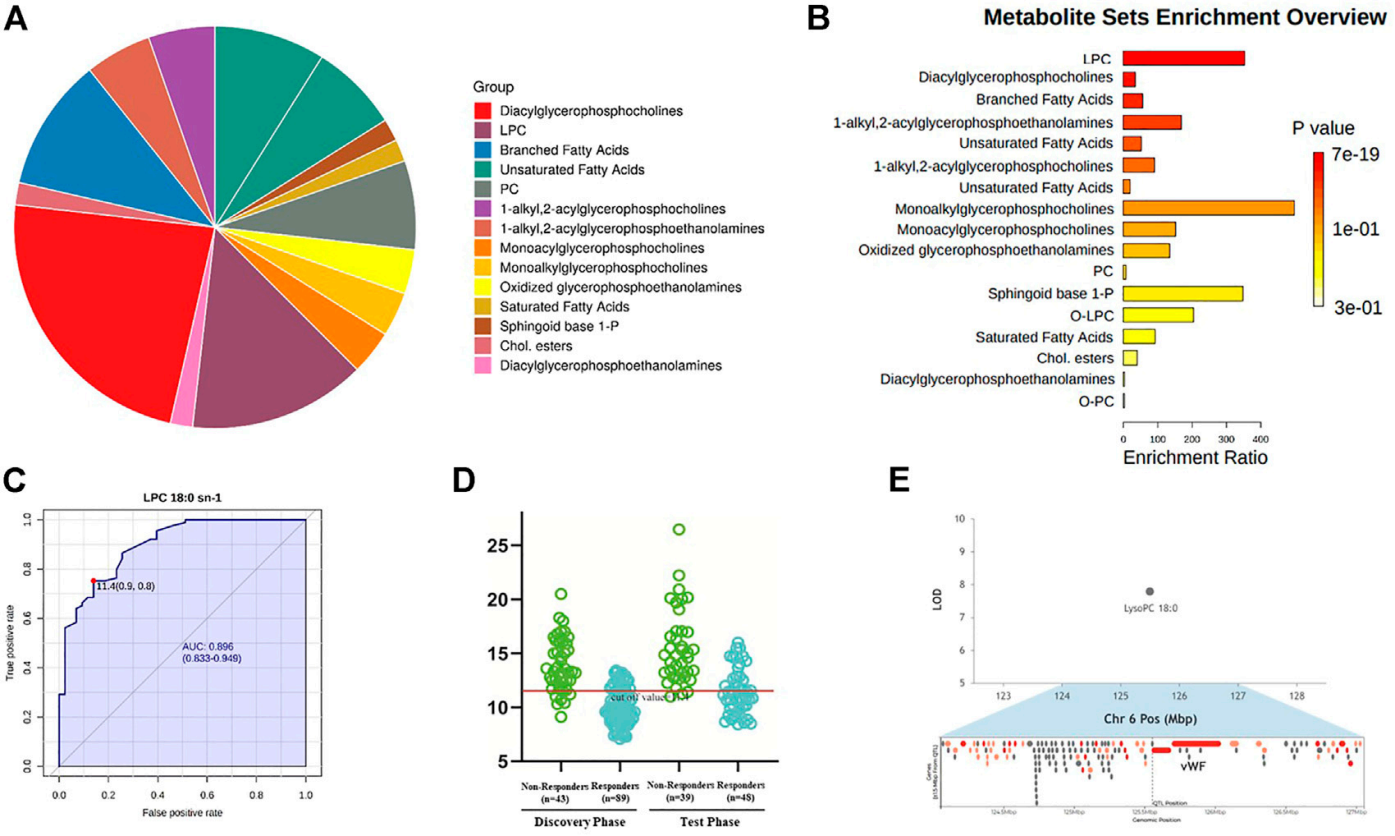
Enriched metabolite pathways and potential biomarker associated with different response to anti-VEGF treatment. **(A)** Interactive pie chart of sub-classes of differential metabolites. **(B)** Bar plot of enriched metabolite pathways. Responders and non-responders differed most significantly in metabolism of LPC (*p* = 7.16 x 10^-19) and diacylglycerophosphocholine (*p* = 6.96 x 10^-17). **(C)** Diagnostic outcome of LPC 18:0 in the discovery group. The area under curve is 0.896 with 95% confidence internal between 0.833 and 0.949, to discriminate responders from non-responders. The optimal cut-off value of LPC 18:0 was 11.4. **(D)** Prediction accuracies by LPC 18:0 in the discovery and validation group. This cut-off value 11.4 was used to predict treatment response in the validation group. The predictive value was 72.4%. **(E)** Hotspot mapping to chromosome 6: 125.554 Mbp indicated the gene associated with LPC 18:0 (highlighted in red). The single nucleotide polymorphism of von Willebrand Factor was highly relevant with the level of LPC 18:0.

The sub-classes of differential metabolites associated with treatment response included diacylglycerophosphocholines, lysophosphatidylcholine (LPC), branched fatty acids, unsaturated fatty acids, phosphocholine (PC), etc (Figure 5A). Responders and non-responders differed most significantly in metabolism of LPC (*p* = 7.16 x 10^-19) and diacylglycerophosphocholine (*p* = 6.96 x 10^-17). The enriched metabolites pathways are listed in Figure 5B, and the detailed information of the pathways is summarized in Supplementary Table S2. Among the differential metabolites, LPC 18:0 exhibited the highest AUC, which is 0.896 with 95% confidence internal between 0.833 and 0.949, to discriminate responders from non-responders (Figure 5C). Prediction accuracies by LPC 18:0 in the discovery and validation groups are illustrated in Figure 5D. The optimal cut-off value of LPC 18:0 in the discovery group was 11.4. This cut-off value was then used to predict treatment response in the validation group, and the predictive value was 72.4%. According to our genome-metabolite connections analysis on the platform Lipid Genie’s Lipid Viewer, the single nucleotide polymorphism (SNP) of von Willebrand Factor (vWF) was highly relevant with the level of LPC 18:0 (Figure 5E).

## Discussion

Anti-VEGF drugs have revolutionized the treatment of retinal neovascular diseases. Conbercept, an anti-VEGF agent developed recently in China, reduced CRT in patients with nAMD in previous clinical studies (Zhang et al., 2011; Li et al., 2014; Liu et al., 2019). Nevertheless, there was still a lack of reliable and accurate methods to predict the efficacy of anti-VEGF treatment. The authors have conducted a prospective longitudinal study (NCT03128463) to investigate metabolic biomarkers related to treatment response to conbercept. The main findings of this study included three aspects.

Firstly, there were no significant differences in serum metabolic profiles before and after intravitreal injection of conbercept. Previous studies revealed that plasma level of VEGF was reduced after injection of aflibercept, but no change was found after ranibizumab treatment in patients with AMD (Wang et al., 2014; Yoshida et al., 2014; Zehetner et al., 2015). However, the influence of anti-VEGF treatment on serum metabolomics has not been reported previously. To our knowledge, our study was the first to disclose that intraocular anti-VEGF administration had little influence on serum metabolism. The diversity of treatment response was not due to the metabolic changes caused by the drug.

Secondly, the authors identified a clear difference of baseline metabolic profiles between responders and non-responders, and inferred that metabolism was one of the factors determining treatment response. Although some metabolic pathways related to the onset and progression of AMD and PCV have been studied these years, there is still no consensus over the effect of metabolomics on treatment response. The well-recognized pathways associated with AMD progression included dysregulation of lipid metabolism, nucleotide metabolism, carbohydrate metabolism, amino acid metabolism, etc (Hou et al., 2020). Interestingly, the differential metabolites we identified were mostly enriched to lipids, including lipids from subclasses of LPC, PC, carnitine, fatty acids, etc. Research on the relationship between lipids and angiogenesis was just beginning. Kananen et al. reported that high level of serum total cholesterol led to the development of AMD (Kananen et al., 2021). Samson et al. utilized chorioallantoic membrane assay of chick embryo and gas chromatography-mass spectrometry analysis to uncover the specific lipids related to angiogenesis. High levels of LPC, lysophosphatidylethanolamine and cholesterol were detected in the vessel area (Samson et al., 2021). We speculated that specific lipids might regulate the retino-choroidal angiogenesis microenvironment, resolve the activity of CNV, and therefore affect anti-VEGF response. Gao et al. implicated that the serum levels of glycerophosphocholine, LysoPC (18:2) and PS (18:0/20:4) were increased in non-responders (Gao et al., 2020), which was consistent with ours.

The most important disorder pathway we identified was LPC metabolism. In a study of patients at different stages of AMD, patients with nAMD demonstrated increased serum levels of total LPC and LPC 18:0, indicating that LPC participated in angiogenesis (Semba et al., 2019). Fundamental experiment showed that LPC was related to endothelial dysfunction via activation of PKC signaling pathway (Zhao et al., 2021). We performed ROC curve analysis to screen potential biomarkers to discriminate responders. LPC18:0 showed the highest diagnostic efficiency to predict treatment response to conbercept at the beginning of the primary phase, but the mechanism needs further research.

Apart from LPC, pathway enrichment analysis also suggested alterations of diacylglycerophosphocholine metabolism, and the principle metabolites of this pathway were PCs. Increased circulating oxidized phospholipids were related to the pathogenesis of various diseases (Karki et al., 2020). The function of PCs in eye diseases was deemed to reflect oxidative stress of lipids (Cabrerizo et al., 2017a; Cabrerizo et al., 2017b). PCs were identified as discriminating metabolites of AMD (Kersten et al., 2019) and PCV (Li et al., 2016), and our results suggested that PCs could be biomarkers to predict treatment response. Another important metabolite identified was carnitine C 14:3, and the accumulation of carnitine might have resulted from disorders of carnitine metabolism (Knottnerus et al., 2018). The major function of carnitine was to shuttle long-chain fatty acids into mitochondrial matrix (Almannai et al., 2019). Recent research revealed that the carnitine shuttle pathway was more significantly changed in nAMD than intermediate AMD (Mitchell et al., 2021), implying its role in CNV generation.

Thirdly, the authors identified genetic variants associated with differential metabolite through public data analysis, with the purpose of clarifying genome-metabolite connections. SNP of vWF was closely associated with LPC 18:0. Despite the main function of regulating bleeding disorder, vWF was one of the specific surface markers of endothelial cells (Lagarkova et al., 2008). Immunohistochemistry of CNV membranes and polypoidal vessels of human eyes verified that vWF was expressed in the vascular endothelial cells (Wakusawa et al., 2008). Yamashita et al. reported elevated plasma levels of vWF:Antigen in patients with AMD, elucidating the participation of vWF in AMD (Yamashita et al., 2018). Nevertheless, how vWF regulates LPC 18:0 needs further investigation.

Honestly, there are some limitations to this study. (i) The relative small sample size of the study population, which could influence repeatability of the results. Hence, the authors recruited 87 independent subjects from other 12 clinical sites as the validation group to verify the main findings. (ii) According to the protocol of the study, only patients receiving conbercept treatment were enrolled, which could lead to bias. Therefore, the results should be objectively interpreted, and further research is required to elucidate whether our results can be expanded to other anti-VEGF drugs. (iii) The time point to define treatment response was 1 month after the first injection. Although ‘3 + PRN’ regimen was recommended for nAMD based on the results of randomized controlled clinical trials, many patients could not afford three loading doses. Thus, it is critical to develop metabolic biomarkers to predict response at the beginning of the treatment. More longitudinal studies are needed to clarify the relationship between metabolites and response to anti-VEGF treatment in the primary phase as well as in the maintained phase.

## Conclusion

Responders and non-responders treated with conbercept differed most significantly in metabolism of LPC and diacylglycerophosphocholine. LPC 18:0 could be a potential biomarker to discriminate responders. Further research is needed to confirm the relationship between lipids and angiogenesis.

## Data Availability

The raw data supporting the conclusions of this article will be made available by the authors, without undue reservation.
